# Oncogenic long intervening noncoding RNA Linc00284 promotes c-Met expression by sponging miR-27a in colorectal cancer

**DOI:** 10.1038/s41388-021-01839-w

**Published:** 2021-05-28

**Authors:** Jun You, Jiayi Li, Chunlin Ke, Yanru Xiao, Chuanhui Lu, Fakun Huang, Yanjun Mi, Rongmu Xia, Qiyuan Li

**Affiliations:** 1 Department of Gastrointestinal Oncology Surgery, Cancer Center, The First Affiliated Hospital of Xiamen University, Xiamen, Fujian PR China; 2 The School of Clinical Medical, Fujian Medical University, Fuzhou, Fujian PR China; 3 Department of Medical Oncology, Xiamen Key Laboratory of Antitumor Drug Transformation Research, The First Affiliated Hospital of Xiamen University, School of Clinical Medicine, Fujian Medical University, Xiamen, Fujian PR China; 4 Department of Radiotherapy, The First Affiliated Hospital of Fujian Medical University, Fuzhou, Fujian PR China; 5 Department of Medical Oncology, The First Affiliated Hospital of Xiamen University, Xiamen, Fujian PR China; 6 Department of Colorectal Cancer Surgery, Xiamen Cancer Center, The First Affiliated Hospital of Xiamen University, Teaching Hospital of Fujian Medical University, Xiamen, Fujian PR China; 7 Department of Gastrointestinal Surgery, The First Affiliated Hospital of Fujian Medical University, Fuzhou, Fujian PR China; 8 Department of Medical Oncology, Xiamen Key Laboratory of Thoracic Tumor Diagnosis and Treatment, Institute of Lung Cancer, The First Affiliated Hospital of Xiamen University, School of Clinical Medicine, Fujian Medical University, Xiamen, Fujian PR China; 9 School of Medicine, Xiamen University, Xiamen, Fujian PR China; 10 National Institute of Data Science in Health and Medicine, School of Medicine, Xiamen University, Xiamen, Fujian PR China

**Keywords:** Colorectal cancer, siRNAs

## Abstract

Emerging evidences suggest that long noncoding RNA (lncRNA) plays a vital role in tumorigenesis and cancer progression. Here, the aim of this study is to investigate the biological function of long intervening noncoding RNA Linc00284 in colorectal cancer (CRC). The expression levels of Linc00284, miR-27a and c-Met were evaluated by qPCR and/or Western blotting. Immunohistochemistry was used to detect the expression of Ki67 and Phh3 in tumor tissues. The interaction between Linc00284, miR-27a and c-Met was validated by luciferase reporter assay and RNA immunoprecipitation (RIP) assay. Cell function experiments, including CCK-8, wound-healing and transwell invasion assays, were conducted. The in vivo studies were performed with the subcutaneous tumor xenograft mouse models. Our findings reveal that Linc00284 is upregulated in CRC tissues and colorectal cancer cell lines HCT116 and SW480 in comparison with corresponding para-carcinoma tissues and human fetal colonic mucosa cells FHC. High expression of Linc00284 in tumor tissues is associated with tumor metastasis and predicts a poor clinical outcome in CRC patients. Serum Linc00284 is increased, while miR-27a is decreased in CRC patients compared to healthy controls. ROC curve analysis indicates that serum Linc00284 and miR-27a produce the area under the curve (AUC) value of at 0.8151 and 0.7316 in patients with colorectal cancer compared to healthy individuals, respectively. Additionally, results in vitro and in vivo experiments suggest that Linc00284 silencing significantly suppresses CRC cell proliferation and/or invasion. Mechanistically, Linc00284 promotes c-Met expression by acting as miR-27a sponge, leading to the activation of downstream signaling pathways, thereby causing malignant phenotypes of CRC cells. Taken together, Linc00284 exhibits oncogenic function and the disturbance of Linc00284/miR-27a/c-Met regulatory axis contributes to CRC progression, providing new insight into the pathogenesis of colorectal cancer. Importantly, the expression levels of serum Linc00284 and miR-27a may serve as clinical biomarkers for CRC diagnosis.

## Introduction

Colorectal cancer (CRC) is one of the most common malignancies worldwide [1]. Although the therapeutic effect of surgical resection with adjuvant chemoradiotherapy or targeted therapy in CRC has been greatly improved during the past decades [2], the prognosis of patients with advanced CRC is still very poor [3]. Therefore, it is particularly important to find effective biomarkers and targets for the early diagnosis and treatment of CRC.

Long noncoding RNA (lncRNA) is a class of RNA longer than 200 nucleotides and lacks a complete open reading frame (ORF), including LincRNA [4–6]. Increasing studies have shown that LincRNAs are abnormally expressed in various types of cancers, including colorectal cancer [5, 7], where LincRNA acts as a tumor suppressor or oncogene, inhibiting or promoting tumorigenesis and cancer progression. For instance, linc01503 and linc-ITGB1 promote CRC progression and metastasis [8, 9]. Linc00284 is a newly discovered LincRNA. Previous work has showed that Linc00284 is implicated in angiogenesis during ovarian cancer development by recruiting NF-κB1 and down-regulating mesoderm-specific transcript (MEST) [10]. Furthermore, new evidence suggested that Linc00284 was involved in the progression of gastric cancer [11], primary papillary thyroid carcinoma [12], breast cancer [13], and hepatocellular carcinoma [14]. However, the underlying molecular mechanism of Linc00284 in CRC remains unclear.

LncRNA-miRNA-mRNA network plays an important regulatory role in the pathogenesis of CRC [15], of which microRNAs (miRNAs) are endogenous noncoding RNAs with approximately 22 nucleotides and can bind to the 3′ untranslated region (3′UTR) of target messenger RNA (mRNA), regulating gene expression [16, 17]. miRNAs are broadly involved in various pathological processes, including tumor initiation and progression [18–20]. Recent work in CRC has suggested that miR-27a facilitates colorectal cancer progression via activation of Wnt/β-catenin pathways [21, 22]. Additionally, it is worth noting that lincRNA, including Linc00284, regulates gene expression by competitively binding endogenous miRNA [5, 7]. In present study, we aim to investigate the biological functions of Linc00284 in colorectal cancer. Our findings demonstrated that targeting Linc00284 holds considerable promise for clinical anticancer therapy.

## Results

### Linc00284 is overexpressed in CRC and high Linc00284 expression predicts poor outcome in patients with colorectal cancer

**Fig. 1:**
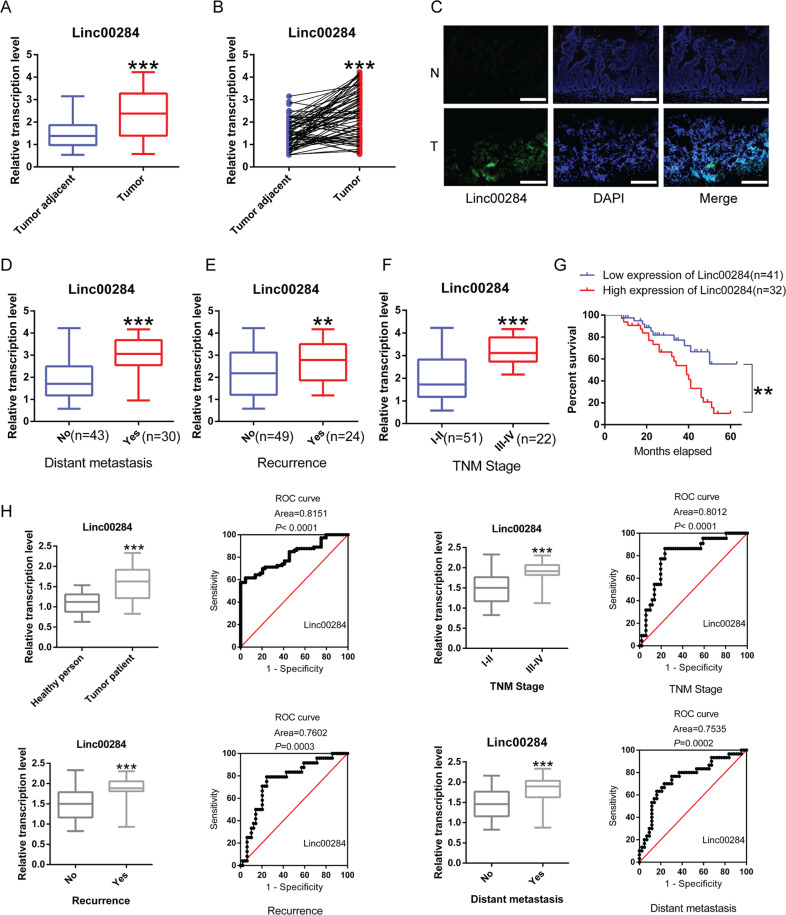
Linc00284 overexpression is associated with the clinicopathological characteristics and poor prognosis in patients with colorectal cancer. **A**, **B** Linc00284 expression in 73 human CRC samples and paired adjacent normal tissues. **C** The expression of Linc00284 in CRC and adjacent normal tissues was detected by immunofluorescence. Scale bar, 100 μm. **D**–**F** The expression level of Linc00284 was significantly higher in tumor tissues of (**D**) patients with metastatic CRC than those without metastasis or (**E**) patients with recurrent CRC compared with those without recurrent CRC or (**F**) patients with stage I-II CRC than those with stage III-IV CRC. **G** Kaplan–Meier curve of overall survival. **H** The level of serum Linc00284 in patients with CRC and healthy controls was measured by qPCR analysis. Data are presented as the mean ± S.D. from three independent experiments. *P* values were calculated using two-sided paired *t*-test. **P < 0.01; ***P < 0.001.

In this study, qPCR analysis revealed that the expression of Linc00284 was significantly upregulated in colorectal cancer tissues compared with adjacent normal tissues (*N* = 73, Fig. 1A, B). Identical results were obtained by immunofluorescence (Fig. 1C). Besides, Linc00284 overexpression was associated with cancer metastasis (Fig. 1D), relapse (Fig. 1E), TNM staging (Fig. 1F) and poor overall survival (Fig. 1G). Additionally, Linc00284 level was increased in the serum of patients with colorectal cancer and significantly associated with TNM stage, tumor recurrence and distant metastasis (Fig. 1H). ROC curve analysis indicated that the area under the ROC curve (AUC) value of Linc00284 level reached at 0.8151 in the peripheral blood serum of patients with colorectal cancer (*n* = 73), as compared with the healthy controls (*n* = 43). These data suggested that Linc00284 may play an important role in CRC progression and serum Linc00284 level in patients with colorectal cancers has been suggested to be a useful biomarker for cancer diagnosis and prognosis.

### Linc00284 knockdown represses CRC cell proliferation and growth in vitro and in vivo

**Fig. 2:**
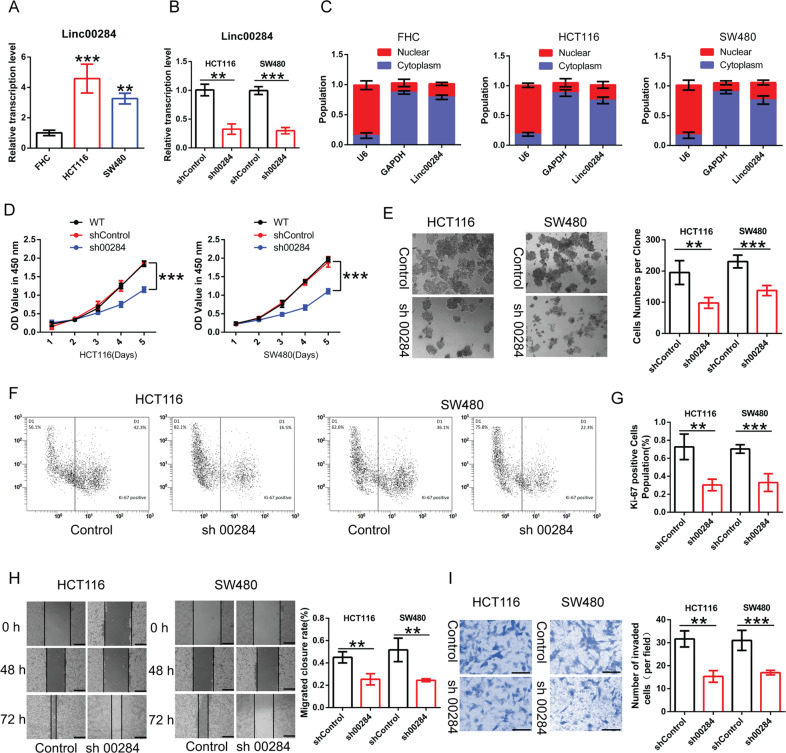
Linc00284 knockdown represses CRC cell proliferation, migration and invasion in vitro. **A** Linc00284 expression in normal colon epithelial cells FHC, HCT116 and SW480 cells was determined by qPCR analysis. **B** Linc00284 silencing efficiency of HCT116 and SW480 cells was verified by qPCR analysis. **C** The levels of nuclear Linc00284 and cytosolic Linc00284 were evaluated by qPCR analysis after separating the nucleoplasm from the cytoplasm. **D** The cell viability of Linc00284-silenced CRC cells and the control cells was measured using Cell Counting Kit-8 (CCK-8) assay. **E** Colony formation assays was performed to test cell proliferative ability. **F** Proliferation of cancer cells was analyzed with Ki67 staining by flow cytometry. **G** Quantification of Ki67 staining by flow cytometry. **H** The migration of Linc00284-silenced CRC cells was detected by wound healing assay. **I** The invasion of Linc00284-silenced CRC cells was analyzed by transwell assay. Data are presented as the mean ± S.D. from three independent experiments. Comparisons between two groups were analyzed using two-sided unpaired *t-*test. Multiple comparisons were analyzed using one-way analysis of variance (ANOVA), post-hoc least significant difference (LSD) test. **P < 0.01; ***P < 0.001. sh00284 lentiviral vector-mediated Linc00284 silencing by short hairpin RNA.

To understand the role of Linc00284 in CRC cells, the expression of Linc00284 in normal colonic epithelial cells FHC and CRC cells HCT116 and SW480 was detected by qPCR analysis. Results showed that Linc00284 expression in HCT116 and SW480 cells was much higher than that in FHC cells (Fig. 2A). Further, Linc00284-knockdown HCT116 and SW480 cell lines were established and qPCR analysis confirmed that Linc00284 expression was significantly downregulated in two types of cells in comparison to the control cells (Fig. 2B). After extraction of the nucleus and cytoplasm, we found that Linc00284 was mainly localized in the cytoplasm of FHC, HCT116 and SW480 cells (Fig. 2C). CCK-8 assay showed that there was no significant difference in cell viability of sh-Control cells and wild-type (WT) cancer cells; however, the cell viability of Linc00284-knockdown CRC cells significantly reduced compared to the control cells (Fig. 2D). Moreover, colony formation assay and Ki-67 staining confirmed that the proliferative capability of Linc00284-knockdown CRC cells was significantly suppressed compared to the control cells (Fig. 2E–G). Additionally, wound healing and transwell invasion assay showed that Linc00284 knockdown strikingly attenuated the migratory and invasive abilities of HCT116 and SW480 cells (Fig. 2H, I). Conversely, Linc00284 overexpression promoted the malignant phenotypes of CRC cells (Supplementary Fig. S1).

**Fig. 3:**
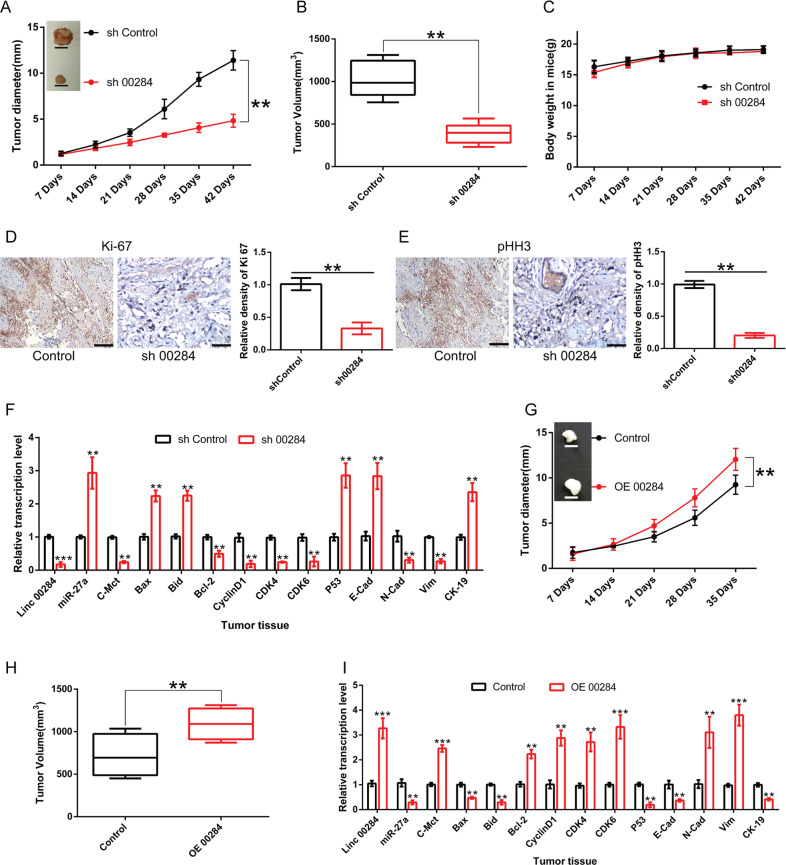
Linc00284 knockdown inhibits CRC cell growth in vivo. **A** Tumor diameter in the Linc00284-silenced group and the Control group (*N* = 6 mice per group). **B** Quantitative of xenograft tumor volume. **C** Body weight of all mice. **D**, **E** Immunohistochemistry was conducted to detect the expression levels of Ki-67 and pHH3 in tumor tissues and adjacent normal samples. **F** The mRNA expression of Linc00284, miR-27a, *c-Met, Bax, Bcl-2, CyclinD1, CDK4, CDK6, P53, E-Cadherin, N-Cadherin, Vimentin* and *Cytokeratin-19* in tumor tissues with silencing of Linc00284 and control samples. **G** Tumor diameter in the Linc00284-overecpressed group and the control group (*N* = 6 mice per group). **H** Tumor volume in xenograft mouse model with/without overexpression of Linc00284. **I** The mRNA expression of Linc00284, miR-27a, c-Met, *Bax, Bcl-2, CyclinD1, CDK4, CDK6, P53, E-Cadherin, N-Cadherin, Vimentin* and *Cytokeratin-19* in tumor tissues with overexpression of Linc00284 and the control samples. Data are presented as the mean ± S.D. from three independent experiments. *P* values were calculated using two-sided unpaired *t*-test. **P < 0.01; ***P < 0.001. E-cad E-Cadherin, N-Cad N-Cadherin, CK-19 Cytokeratin-19, sh00284 lentiviral vector-mediated Linc00284 silencing by short hairpin RNA.

To further determine the biological function of Linc00284 in CRC progression, xenograft nude mouse model was used in this study. Our findings demonstrated that the silencing of Linc00284 significantly inhibited tumor growth in vivo without adverse effect on body weight of mice (Fig. 3A–C); meanwhile, the expression levels of cell proliferation markers Ki67 and Phosphohistone-H3 (pHH3) were markedly reduced in Linc00284 knockdown group in comparison to the control group (Fig. 3D, E). Notably, the expression levels of miR-27a, pro-apoptotic gene (*Bax* and *Bid*), tumor suppressor gene *P53*, epithelial markers (*E-Cadherin* and *Cytokeratin19*) were significantly upregulated, whereas the expression levels of oncogene *c-Met*, genes involved in cell cycle progression, including *CDK4*, *CDK6* and *CyclinD1*, mesenchymal markers (*N*-*Cadherin* and *Vimentin*) and anti-apoptotic gene *Bcl-2* were downregulated in tumor tissues (Linc00284 was silenced) compared to the control samples (Fig. 3F). In contrast, the overexpression of Linc00284 promoted CRC cell growth and produced the opposite effects on the above-mentioned gene expressions in tumor tissues (Fig. 3G–I). Together, these findings point to a tumor-promoting role for Linc00284 in CRC.

### Linc00284 binds directly to miR-27a and negatively regulates the expression of miR-27a

**Fig. 4:**
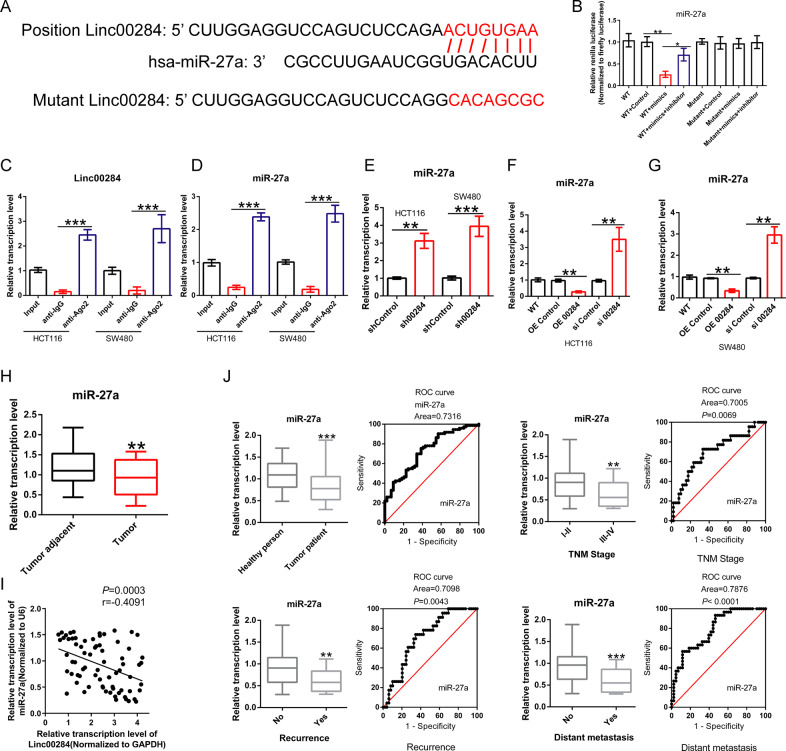
Linc00284 directly binds to miR-27a and negatively regulates miR-27a expression. **A** Schematic diagram of the potential binding sites between Linc00284 and miR-27a. **B** Dual-luciferase reporter assay and **C** RIP experiment confirmed the interaction between Linc00284 and miR-27a. **D** Binding of miR-27a and Ago2 determined by RIP assay. **E** The expression level of miR-27a in Linc00284 knockdown CRC cells. **F** miR-27a level in HCT116 cells transfected with pcDNA Linc00284 vectors or si Linc00284 for 48 h. **G** miR-27a expression in SW480 cells transfected with pcDNA Linc00284 or si Linc00284 for 48 h. **H** miR-27a expression in human CRC tissues was detected by qPCR analysis (*N* = 73). **I** Pearson correlation test suggested a negative correlation between Linc00284 and miR-27a expression. **J** Serum miR-27a level in patients with CRC (*N* = 73) compared to healthy controls (*N* = 43). Data are presented as the mean ± S.D. from three independent experiments. Comparisons between two groups were analyzed using two-sided paired *t*-test. Multiple comparisons were analyzed using one-way analysis of variance (ANOVA), post-hoc least significant difference (LSD) test. *P < 0.05; **P < 0.01; ***P < 0.001; NS not significant difference, sh00284 lentiviral vector-mediated Linc00284 silencing by short hairpin RNA.

LincRNAs act as competing endogenous RNA (ceRNA) of microRNA, thereby regulating the expression of cancer-related genes. To determine the target miRNA of Linc00284, online bioinformatics software (Starbase v3.0) was applied. It was predicted the binding sites between Linc00284 and miR-27a (Fig. 4A). Dual-luciferase reporter assay revealed that miR-27a mimics decreased the luciferase activity of the WT-Linc00284 reporters, but not that of Mutant-Linc00284 in 293T cells, suggesting that Linc00284 directly bound to miR-27a (Fig. 4B). Consistently, RIP analysis determined that both Linc00284 and miR-27a were enriched on anti-Ago2 magnetic beads (Fig. 4C, D). FISH assay indicated that both Linc00284 and miR-27a were expressed in the cytoplasm of CRC cells (Supplementary Fig. S2). Moreover, Linc00284 knockdown contributed to the up-regulation of miR-27a (Fig. 4E).

To further unravel the biological functions of Linc00284 and miR-27a, HCT116 and SW480 cells were transfected with pcDNA-00284, the small interfering RNA targeting Linc00284 (si-Linc00284) or their corresponding control vectors. We found that the silencing efficiency of si-Linc00284 could maintain 5 days (Supplementary Fig. S3). When CRC cells were transiently silenced Linc00284 by si-Linc00284, miR-27a expression was markedly elevated, whereas enforced expression of Linc00284 resulted in decreased miR-27a level in CRC cells (Fig. 4F, G). qPCR analysis further suggested that miR-27a expression was significantly downregulated in human CRC tissues (Fig. 4H) and negatively correlated with Linc00284 expression (Fig. 4I). These findings indicated that Linc00284 targeted directly to miR-27a and negatively regulated miR-27a expression.

miR-27a has proved to be a useful diagnostic and/or prognostic biomarker in various types of solid cancers [23]. In this study, the level of miR-27a in serum samples from patients with colorectal cancer (*n* = 73) was strikingly decreased as compared to the healthy individuals (*n* = 43, Fig. 4J). Besides, the level of miR-27a rises in serum correlated with the TNM stage, tumor recurrence and metastasis.

Given that miR-27a and miR-27b are two highly homologous microRNAs, we implemented the dual-luciferase reporter assay against miR-27b as described above, and the results confirmed that the addition of exogenous miR-27b mimics significantly reduced the luciferase activity in WT-Linc00284 (not Mutant-Linc00284) vectors-transfected 293T cells, indicating that miR-27b was also directly bind to Linc00284 (Supplementary Fig. S4A). However, RIP assay did not yield the same results (Supplementary Fig. S4B-C). To explain the difference between these two experiments, we examined the expression levels of both miR-27a and miR-27b in colorectal cancer cells, and data showed that miR-27a expression was much higher than that of miR-27b, which might be the reason why it could not be detected by RIP assay (Supplementary Fig. S4D). In the subsequent cellular functional experiments, we found that exogenous miR-27b mimics in CRC cells significantly inhibited CRC cell proliferation, migration and invasion, while miR-27b inhibitors did not have such an effect (Supplementary Fig. S4E-G). Taken together, although our findings suggest that miR-27b also binds to Linc00284, miR-27b is expressed in colorectal cancer cells at a much lower level than miR-27a. Hence, miR-27a is considered as the main downstream target of Linc00284 in this study.

### Linc00284 facilitates the malignant behaviors of CRC cells by targeting miR-27a

**Fig. 5:**
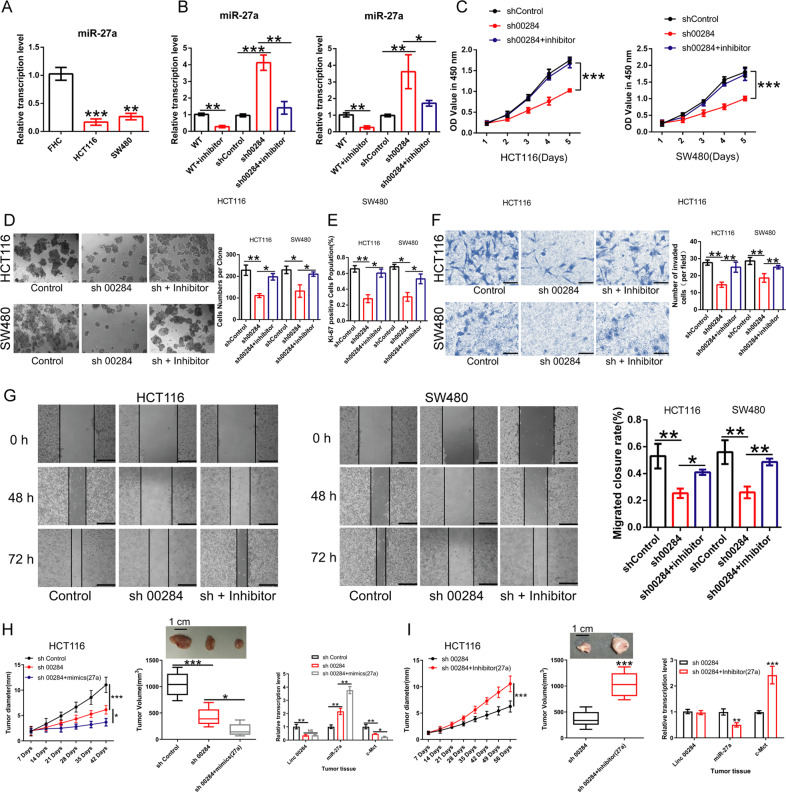
Linc00284 suppresses the proliferation, migration and invasion of CRC cells by targeting miR-27a. **A** miR-27a expression in normal colonic epithelial cells FHC and CRC cell lines (HCT116 and SW480) was detected by qPCR analysis. **B** The effect of miR-27a inhibitor on the expression of miR-27a in Linc00284 knockdown CRC cells. Left, HCT116 cells; right, SW480 cells. **C** The effect of miR-27a inhibitor on CRC cell proliferation was measured by CCK-8 assay. Left, HCT116 cells; right, SW480 cells. **D** The effect of miR-27a inhibitor on the proliferation of Linc00284 knockdown CRC cells was determined by colony formation assay. **E** Ki-67 staining was used to test the effect of miR-27a inhibitor on the proliferation of Linc00284 knockdown CRC cells. **F** The migration ability of CRC cells was measured by wound healing assay. **G** The invasive ability of CRC cells was monitored by transwell assay. Xenograft tumors model for effect of Linc00284 knockdown alone or in combination with miR-27a (**H**) mimics/(**I**) inhibitor treatment on the growth of HCT116 xenografts. Tumor growth was monitored by measurements of tumor diameter using calipers (left); the tumor volume (middle); the expression of Linc00284, miR-27a and c-Met in tumor tissues (right). Data are presented as the mean ± S.D. from three independent experiments. *P* values were calculated using one-way analysis of variance (ANOVA), post-hoc least significant difference (LSD) test. *P < 0.05; **P < 0.01; ***P < 0.001. sh 00284, lentiviral vector-mediated Linc00284 silencing by short hairpin RNA.

To determine the effect of the Linc00284/miR-27a regulatory axis on CRC cells, the proliferative and invasive capabilities of CRC cells were measured by CCK-8, cell colony formation, transwell assays. We found that miR-27a expression in CRC cells (HCT116 and SW480) was significantly lower than that in normal colonic epithelial cells FHC (Fig. 5A). Next, HCT116 and SW480 cells with stably silencing Linc00284 were transfected with exogenous miR-27a mimics or inhibitor. The transfection efficiency was determined by qPCR analysis (Fig. 5B). CCK-8 (Fig. 5C) and colony formation assays (Fig. 5D) revealed that the down-regulation of miR-27a rescued the inhibitory effect of linc00284 knockdown on CRC cell proliferation. Similar results were obtained in Ki-67 staining (Fig. 5E). Wound healing (Fig. 5F) and transwell invasion assays (Fig. 5G) uncovered that miR-27a inhibitor could partially reverse the inhibition of cell migratory and invasive abilities caused by Linc00284 knockdown. Further, the effect of Linc00284/miR-27a regulatory axis on tumor growth was verified by subcutaneous tumor xenograft mouse models. Results confirmed that Linc00284 knockdown could significantly reduce cell proliferation in vivo compared to the control group. miR-27a mimics treatment in combination with Linc00284 knockdown displayed a synergistic inhibitory effect on tumor cell growth in comparison to the Linc00284 knockdown group. Besides, as compared with the Linc00284 knockdown group, miR-27a inhibitor treatment was able to reverse the reduced proliferative capacity of CRC cells caused by Linc00284 knockdown (Fig. 5H, I). These in vivo studies corroborated our in vitro findings and indicated that Linc00284 promoted the malignant behaviors of CRC cells in vitro and tumor growth in vivo by targeting miR-27a.

### c-Met is a direct target of miR-27a and Linc00284 regulates c-Met expression by regulating miR-27a

**Fig. 6:**
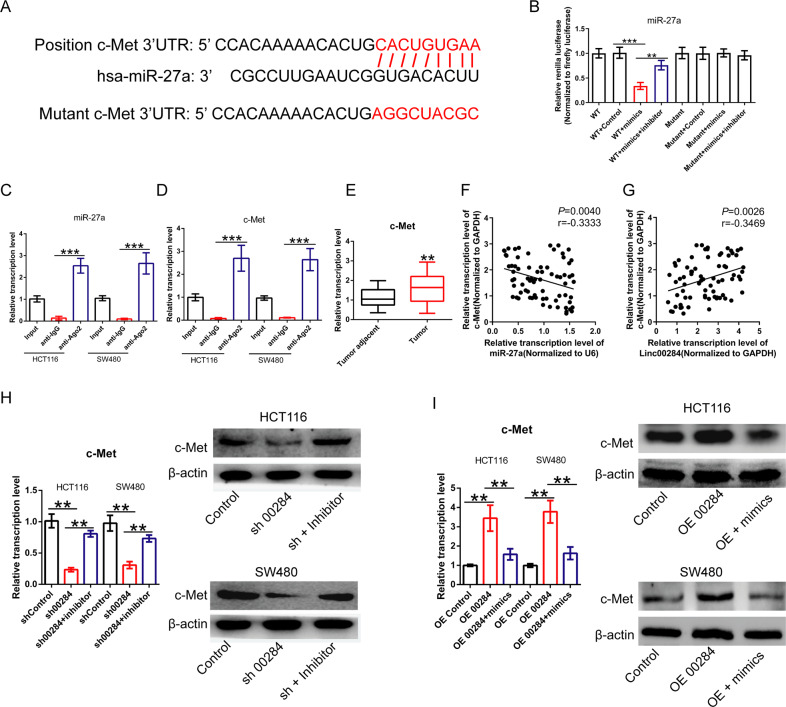
Linc00284 regulates c-Met expression by adsorbing miR-27a. **A** Schematic diagram of the binding sites between miR-27a and 3′UTR of c-Met mRNA. **B** The luciferase activity was tested after co-transfection with wild type (WT) 3′UTR of c-Met mRNA/mutant 3′UTR of c-Met mRNA and miR-27a mimics for 48 h. RIP assay was used to determine the interaction between (**C**) miR-27a and (**D**) c-Met in CRC cells. **E** The expression of c-Met in 73 paired CRC tissues and adjacent tissues was tested by qPCR analysis. **F** Pearson correlation analysis showed the correlation between c-Met and miR-27a expression. **G** The correlation between c-Met and Linc00284 expression. **H** The effects of Linc00284 knockdown or in combination with miR-27a inhibitor treatment for 48 h on the mRNA and protein expression of c-Met in HCT116 cells. **I** The effects of Linc00284 overexpression or in combination with miR-27a mimics treatment for 48 h on the mRNA and protein expression of c-Met in HCT116 cells. Data are presented as the mean ± S.D. from three independent experiments. Comparison between two groups were analyzed using two-sided paired student’s *t*-test. Multiple comparisons were analyzed using one-way analysis of variance (ANOVA), post-hoc least significant difference (LSD) test. **P < 0.01; ***P < 0.001; NS not significant difference, sh00284 lentiviral vector-mediated Linc00284 silencing by short hairpin RNA, OE 00284 overexpression of Linc00284.

By searching on Target Scan [24] (http://www.targetscan.org/vert_72/), it predicted c-Met as a potential target for miR-27a (Fig. 6A). Dual-luciferase reporter assay validated the binding sites between miR-27a and the 3′UTR of c-Met mRNA (Fig. 6B). RIP assay further confirmed the interaction between miR-27a and c-Met (Fig. 6C, D). Besides, the mRNA expression of *c-Met* gene in 73 CRC tissues was significantly upregulated compared to the matched adjacent normal tissues (Fig. 6E). Pearson correlation analysis showed a negative correlation between the expression of c-Met and miR-27a level in CRC samples (Fig. 6F). Conversely, c-Met expression was positively correlated with Linc00284 expression level in CRC tissues (Fig. 6G). Furthermore, c-Met expression was strikingly reduced in Linc00284 knockdown CRC cells, but miR-27a inhibitor partially reversed inhibition of c-Met expression induced by Linc00284 silencing at the transcriptional and translational levels (Fig. 6H). To the contrary, Linc00284 overexpression resulted in the up-regulation of c-Met expression, whereas miR-27a mimics treatment partially reversed c-Met expression caused by Linc00284 overexpression (Fig. 6I). Meanwhile, the expression of c-Met and its downstream molecules were monitored by qPCR analysis. Data showed that overexpression of Linc00284 in CRC cells significantly upregulated the mRNA expression of genes involved in cell cycle progression, including *CDK4*, *CDK6* and *CyclinD1*, mesenchymal markers (*N*-*Cadherin* and *Vimentin*) and anti-apoptotic gene *Bcl-2*), while the expression levels of pro-apoptotic gene *Bax*, epithelial markers (*E-Cadherin* and *Cytokeratin19*) were downregulated (Supplementary Fig. S5) compared to the control cells; however, transfection with miR-27a mimics partially reversed the expression levels of the above genes in CRC cells with Linc00284 overexpressing. In contrast to the above results, Linc00284 knockdown yielded the opposite results in CRC cells; furthermore, miR-27a inhibitor partially reversed the changes caused by Linc00284 knockdown (Supplementary Fig. S6). Overall, Linc00284 exhibits pro-tumor function in CRC by targeting miR-27a/c-Met axis.

### Linc00284/miR-27a/c-Met axis is implicated in CRC progression

**Fig. 7:**
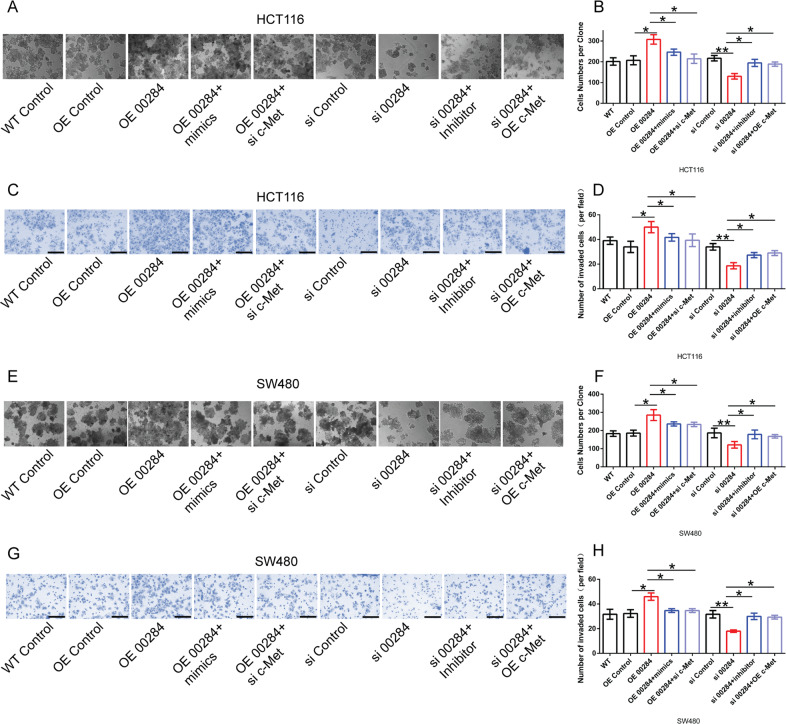
The role of Linc00284/miR-27a/c-Met axis in CRC cells. **A**, **B** The proliferative ability of HCT116 cells were detected by colony formation assay. **C**, **D** The invasive ability of HCT116 cells was measured by transwell assay. **E**, **F** The proliferation of SW480 cells were detected by colony formation assay; **G**, **H** the invasion of SW480 cells was measured by transwell assay. Data are presented as the mean ± S.D. from three independent experiments. Multiple comparisons were analyzed using one-way analysis of variance (ANOVA), post-hoc least significant difference (LSD) test. *P < 0.05; **P < 0.01. si 00284 transient transfection of small interfering RNA to knockdown Linc00284, OE 00284 overexpression of Linc00284, si c-Met transient transfection of small interfering RNA to knockdown c-Met, OE c-Met overexpression of c-Met.

To understand the effect of Linc00284/miR-27a/c-Met axis on CRC cell proliferation and invasion, si-Linc00284, pcDNA3.1-Linc00284 plasmid (overexpression of Linc00284; OE 00284), pcDNA3.1-c-Met plasmid (overexpression of c-Met; OE c-Met), si-c-Met, miR-27a mimics or inhibitor was transfected into CRC cells as indicated in Fig. 7. Our data showed that Linc00284 overexpression could promote the proliferation and invasion of HCT116 cells, while treatment with miR-27a mimics or si-c-Met partially reversed the phenotypes caused by Linc00284 overexpression (Fig. 7A–D). Linc00284 silencing attenuated CRC cell proliferation and invasion, whereas miR-27a inhibitors or pcDNA3.1-c-Met partially reversed the phenotypes induced by Linc00284 silencing (Fig. 7A–D). It was consistent with the results from SW480 cells (Fig. 7E–H). In addition, qPCR and Western blot analysis showed that Linc00284 overexpression significantly elevated *c-Met* expression, while miR-27a mimics or si-c-Met treatment reversed the results caused by Linc00284 overexpression (Supplementary Fig. S7). Conversely, Linc00284 knockdown reduced c-Met expression, but miR-27a inhibitor or pcDNA3.1 c-Met treatment partially reversed the results induced by Linc00284 knockdown (Supplementary Fig. S7). These results suggested that Linc00284 upregulated c-Met expression by acting as miR-27a sponge, thereby promoting CRC progression.

### c-Met inhibitor PHA665752 abolishes the effect of Linc00284 on CRC cells

**Fig. 8:**
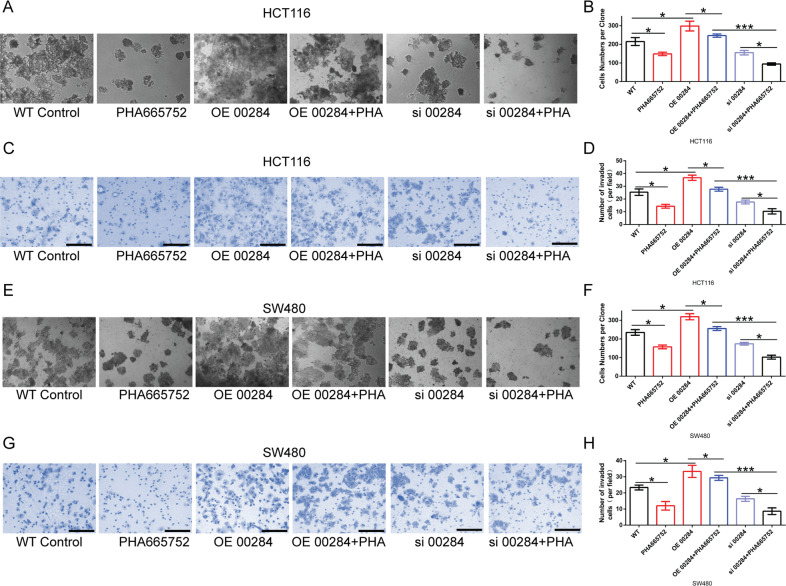
c-Met inhibitor PHA665752 abolishes the effect of Linc00284 on CRC cells. **A**, **B** The proliferative ability of HCT116 cells was tested by colony formation assay. **C**, **D** The invasive ability of HCT116 cells was detected by transwell assay. **E**, **F** The proliferation of SW480 cells was evaluated by colony formation assay. **G**, **H** The invasion of SW480 cells was measured by transwell assay. Linc00284-knockdown/overexpressing CRC cells treated with c-Met inhibitor PHA665752 (30 ng/ml) for 48 h. Data are presented as the mean ± S.D from three independent experiments. Multiple comparisons were analyzed using one-way analysis of variance (ANOVA), post-hoc least significant difference (LSD) test. *P < 0.05, ***P < 0.001. si 00284 transient transfection of small interfering RNA to knockdown Linc00284, OE 00284 overexpression of Linc00284.

To determine the effect of c-Met pharmacological inhibitor PHA665752 on the biological function of Linc00284 in CRC cells, the in vitro assays were performed. Our findings showed that overexpression of Linc00284 significantly enhanced CRC cell proliferative and invasive abilities, but silencing of Linc00284 inhibited CRC cell proliferation and invasion (Fig. 8), which was consistent with the above-mentioned results. PHA665752, a selective small molecule inhibitor of c-Met, represses cell proliferation and invasion (Fig. 8). Further results demonstrated that PHA665752 could effectively counteract the effect of Linc00284 overexpression on cell proliferation and invasion (Fig. 8). PHA665752 treatment in combination with Linc00284 knockdown synergistically inhibited CRC cell proliferation and invasion (Fig. 8). Moreover, it was confirmed by qPCR and Western blot analysis that the silencing of Linc00284 resulted in a significant decrease in c-Met expression, and further treatment with PHA665752 had no effect on the expression of c-Met. Linc00284 overexpression facilitated the expression of c-Met, and further treatment with PHA665752 also had no effect on the expression of c-Met (Supplementary Fig. S8). Hepatocyte growth factor (HGF) and its receptor C-met play a critical role in cancer development and progression. In this study, HGF treatment significantly promoted the proliferation of CRC cells and pcDNA3.1-Linc00284 plasmid (OE-Linc00284) transfection further enhanced the proliferation of CRC cells; however, si-Linc00284 transfection markedly reduced cell proliferative ability (Supplementary Fig. S9). Altogether, Linc00284 exerts its antitumor effects by modulating c-Met expression in CRC.

## Discussion

It has been shown that the dysregulation of LincRNA plays an important role in the development and progression of malignant tumors including colorectal cancer [5, 7, 25]. Here, long intervening noncoding RNA Linc00284, a newly discovered LincRNA, is upregulated and promotes tumor progression in various types of malignancy such as breast tumors and hepatocellular carcinoma [12–14]. The ectopic overexpression of Linc00284 promotes the proliferation, invasion, migration and angiogenesis of ovarian cancer cells, and negatively regulates the activation of apoptosis-related pathways [10]. Consistent with these evidences, our findings indicate that Linc00284 is significantly upregulated in CRC tissues and cell lines. High expression of Linc00284 is positively correlated with metastasis, recurrence, tumor stage and unfavorable prognosis in patients with colorectal cancer. In addition, Linc00284 knockdown can hinder the proliferative ability and migration activity of CRC cells in vitro, and inhibits tumor growth in vivo. These findings elucidate the oncogenic function of Linc00284 in CRC.

Previously, bioinformatics analysis indicated that Linc00284 was a potential target for therapeutic intervention in gastric cancer [11]. Work in primary papillary thyroid cancer (PTC) showed that the expression level of Linc00284 has a negative correlation with the overall survival of PTC patients [12]. In addition, Linc00284 is implicated in angiogenesis of ovarian cancer by recruiting NF-κB1 and suppressing the expression of mesoderm-specific transcript (MEST) [10]. Moreover, a biofunctional analysis reveals that the Linc00284-related ceRNA network is related to serous ovarian carcinoma (SOC) carcinogenesis [26]. In present study, Linc00284 was greatly upregulated in colorectal cancer tissues compared with adjacent normal tissues. Linc00284 overexpression was significantly associated with clinicopathological features, including cancer metastasis, relapse, TNM staging, and poor overall survival of CRC patients. Further, bioinformatics predicted that the binding sites between Linc00284 and miR-27a that has been proved to have inhibitory effects in a variety of human cancers, including gastric cancer [27], lung cancer [28], and CRC [21]. Meanwhile, luciferase reporter and RIP analysis verified the endogenous interaction between Linc00284 and miR-27a. A negative correlation between the expression of Linc00284 and miR-27a was observed. Linc00284 acting as miR-27a sponge promotes CRC cell proliferation and invasion.

c-Met is a hepatocyte growth factor receptor with tyrosine kinase activity [29, 30]. Previous studies have shown that c-Met as an oncogene is upregulated in a variety of cancers, and promotes cancer initiation and progression [31, 32]. c-Met is activated by many molecules, including HGF that initiates transcription of a series of gene expressions, leading to tumor cell growth and invasion [30, 33]. Consistent with these findings, our study demonstrated that c-Met expression was significantly upregulated in CRC tissues in comparison to the adjacent normal tissues. And we confirmed that the interaction between miR-27a and the 3′UTR of c-Met mRNA. Pearson analysis showed that there was a negative correlation between the expression of c-Met and miR-27a in CRC samples. In contrast, c-Met was positively correlated with the expression level of Linc00284 in CRC tissues. Linc00284 positively regulates c-Met expression by sponging miR-27a. Importantly, the co-transfection experiments proved that changes in the levels of Linc00284, miR-27a and c-Met are related to the proliferation of CRC cells, implying that Linc00284/miR-27a/c-Met axis is participated in CRC progression. However, given the complexity of tumor microenvironment, there exist other mechanisms involved in regulating the expression of miR-27a, such as methylation modification or regulation of RNA stability [34], which deserves further investigation.

HGF/c-Met signaling plays an important role in promoting tumor angiogenesis, growth and metastasis [35, 36]. Hepatocyte growth factor (HGF) binds to its receptor, c-Met, activating several signaling pathways, including mitogen-activated protein kinase (MAPK)/extracellular signal-regulated kinase (ERK), PI3K/AKT, NF-κB, and STAT3/5 pathways [29]. Therefore, HGF overexpression and overactivation play a key role in tumorigenesis including CRC [29–31]. c-Met specific inhibitor has demonstrated its exciting antitumor activity [37, 38]. In our study, the overexpression of Linc00284 was positively correlated with the expression level of HGF/c-Met signaling-related molecules, while Linc00284 knockdown inhibited the activation of HGF/c-Met signaling. In addition, Linc00284 overexpression can reverse the inhibitory effect of PHA665752 on CRC cells. After treatment CRC cells with recombinant HGF in combination with Lin00284 knockdown or Linc00284 overexpression, we found that Linc00284 level affected the responsiveness of CRC cells to HGF. Overall, our results confirm that Linc00284 is involved in the progression of CRC by controlling HGF/c-Met signaling. Naturally, further studies will be required to fully elucidate the biological mechanisms underlying these associations in CRC.

LncRNAs [39] and microRNAs [40] are emerging as therapeutic opportunities, and also as non-invasive diagnostic or prognostic biomarkers. In present study, serum Linc00284 was increased whereas serum miR-27a was decreased in patients with colorectal cancer as compared with healthy donors, both of which were related to the TNM stage, tumor recurrence and metastasis of CRC. Therefore, the combined assay of serum Linc00284 and miR-27a is valuable in the diagnosis of colorectal cancer. However, an expanded sample size is needed, and the whether the changes of serum Linc00284 and miR-27a are the causes of the CRC, or the results deserve further exploration.

In summary, our findings indicate that Linc00284 acts as miR-27a sponge, thereby up-regulating c-Met expression. LincRNA-associated ceRNA network plays a critical tumor-promoting role in CRC progression via regulation of HGF/c-Met signaling. Targeting Linc00284 may be a potential therapeutic target for CRC. Besides, in peripheral blood, serum levels of Linc00284 and miR-27a may be used as potential diagnostic biomarkers for colorectal cancer.

## Materials and methods

### Tissue specimens

In this study, 73 CRC tissues, paired adjacent non-tumor samples, the peripheral blood of CRC patients (*N* = 73) and healthy donors (*N* = 43; male, 26; female, 17; age 59–78 years; the median age, 65) were obtained from the First Affiliated Hospital of Xiamen University from February 2014 to August 2019. The clinicopathological data of CRC patients are listed in Table 1. All patients haven’t received radiation or chemotherapy before surgery. Tissue samples were frozen immediately in liquid nitrogen after resection until total RNA was extracted. Serum from peripheral blood was isolated and stored at −80 °C for use. Tissues and blood samples were collected with the patient’s and donor’s informed consent. This study was approved by the Medical Ethics Committee of the First Affiliated Hospital of Fujian Medical University.

**Table 1:** The clinicopathological factors of CRC patients (73 cases).

Characteristics	Number of cases (%)
Age (year)	
≤60	26 (35.62)
>60	47 (64.38)
Gender	
Male	38 (52.05)
Female	35 (47.95)
Tumor-Node-Metastasis stage	
I + II	51 (69.86)
III	22 (30.14)
Distant metastasis	
M0	43 (58.90)
M1 + M2	30 (41.10)
Recurrence	
Yes	49 (67.12)
No	24 (32.88)

### Cell culture

CRC cell lines (HCT116 and SW480 cells), human fetal colonic mucosa cells FHC and 293T cells used in this study were purchased from the American Type Culture Collection (Rockville, MD, USA). The cell lines were authenticated by STR profiling and were mycoplasma free. CRC cells and 293T cells were cultured in Dulbecco’s modified Eagle’s medium (DMEM) (Gibco, Thermo Fisher Scientific Inc., Waltham, MA, USA) containing 10% fetal bovine serum (FBS; Gibco) and 100 U/ml penicillin/streptomycin (Gibco). FHC cells were maintained in DMEM/F12 medium (Gibco) with cholera toxin (10 ng/ml), Hepes (10 mM), insulin (10 mM), transferrin (10 mM), hydrocortisone (100 ng/ml), 10% FBS and penicillin/streptomycin (100 U/ml). Cell culture conditions were as below: 37 °C, 95% humidity and 5% carbon dioxide.

### Plasmid construction and cell transfection

The full-length of linc00284 sequence, the small hairpin RNA of linc00284 (sh 00284) and the small interfering RNA of c-Met (si c-Met), miR-27a mimic and its negative control, miR-27a antagomir and its negative control were purchased from Genechem (Shanghai Genechem Co., Ltd., China). The small hairpin RNA of linc00284 (sh 00284) was cloned into pLVX vectors (Shanghai Genechem Co., Ltd., China) following co-transfection with the packaging plasmids (pMDLg/pRRE, pVSVG and pRSV-REV; Shanghai Genechem Co., Ltd.) into 293T cells to construct lentiviral particles. Seventy-two hours post infection, the cells were selected with 2 μg/ml puromycin for 72 h. Transfection efficiency was evaluated by qPCR after 72 h. Herein, the lentiviral vectors provide a means to express short hairpin RNA (shRNA) to induce stable and long-term Linc00284 silencing in CRC cells. In addition, pcDNA3.1-00284 plasmid (00284), pcDNA3.1-c-Met plasmid (c-Met) and empty plasmid (vector) were constructed. The cells were transfected using lipofectamine 2000 transfection reagent (Thermo Fisher Scientific Inc.) according to the manufacturer’s instructions.

### RNA extraction and qPCR

Trizol reagent was used to extract total RNA according to the instructions (Invitrogen, Thermo Fisher Scientific Inc.). cDNA was synthesized with TaqMan^®^ Reverse Transcription Reagents (CatLog No. N8080234, Thermo Fisher Scientific Inc.). The qPCR reaction was carried out with SuperScript™ IV One-Step RT-PCR System (CatLog No. 12594100, Thermo Fisher Scientific) following manufacturer’s guidance. Gene expression was normalized to GAPDH mRNA expression. Additional Table 1 listed all primers involved in this study. Each experiment was carried out in triplicate and three independent experiments.

### Dual-luciferase reporter assay

According to manufacturer’s instructions, 293T cells were co-transfected with pcDNA3.1 linc00284-wt, pcDNA3.1 linc00284-mut, pcDNA3.1 c-Met-wt, pcDNA3.1 c-Met-mut, miR-27a mimic or control plasmid. After 48 h, total proteins were extracted and luciferase activity was measured using Dual-Luciferase Reporter Assay System (Promega, Madison, Wisconsin, USA).

### RNA immunoprecipitation (RIP) assay

Protein-RNA interactions were identified using RNA immunoprecipitation analysis. Pierce™ Magnetic RNA-Protein Pull-Down Kit (Thermo Fisher Scientific) was used for RIP analysis. The cells were collected and completely lysed in RIP lysis buffer following the manufacturer’s instructions. Next, RIP Lysate was incubated with human anti-Argonaute2 (Ago2) antibody magnetic beads (Millipore, Sigma-Aldrich LLC., St. Louis, MO, USA) or anti-mouse IgG negative control magnetic beads (Millipore). RNA bound with magnetic beads was extracted and verified by RT-qPCR.

### Cell proliferation and colony-formation assays

The cells were plated in 96-well plates. Cell proliferative capability was determined using CCK-8 kit (Beyotime Biotechnology Inc., Shanghai, China) according to the manufacturer’s instructions. Rabbit Anti-Ki67 antibody (Abcam, ab92742, with 1:150 working dilution) was used to assess cell proliferation by flow cytometry. For colony formation assay, the transfected cells were cultured in 6-well plates at 1000 cells per well for 2 weeks. Then, the cells were fixed, stained with 0.1% (W/V) crystal violet, and then photographed under microscope (Olympus, Japan).

### Wound healing and transwell invasion assays

For analysis of cell migration, the cells (5 × 10^6^) were planted into six-well dishes. When the cells reached 90% of confluence, scratched in the cell monolayer using 200 μL sterile tip (1 mm wide). Wound healing rate was monitored using the microscope every 24 h. For the invasion assay, transwell chamber (BD, Franklin Lakes, NJ, USA) pre-coated with Matrigel was used to measure the capacity of cell invasiveness. 5 × 10^4^ cells were seeded into the upper chamber containing serum-free medium. The lower chamber was filled with DMEM culture medium supplemented with 10% FBS. Forty-eight hours later, invasive cells were stained with crystal violet as described above. Five randomly selected fields per chamber were photographed under the microscope (Olympus, Japan).

### Subcutaneous xenograft mouse model

All animal experiments were conducted in compliance with ethical regulations and approved by the Animal Ethics Committee of the First Affiliated Hospital of Xiamen University. A total of 54 male BALB/c nude mice (6 weeks old, 18–20 g) were purchased from Model Animal Research Center of Nanjing University (Jiangsu, China) and randomly divided into nine groups (N = 6 per group). No blinding of animal experiments was done. Linc00284 knockdown/overexpressing HCT116 cells and the control cells (1 × 10^6^ cells in 100-μl cell suspension per group) were implanted subcutaneously into the flanks of immunodeficient mice (6 mice/group), respectively. Additionally, seven days post-transplantation, miR-27a mimics/inhibitor treatments (10 μg in 50 μl of PBS) were performed intratumorally (i.t.) every three days until the termination of the experiment. Control mice were administrated with the same volume of PBS. Tumor growth was measured every week. On day 35, or on day 42, or on day 56 after tumor implantation, the mice were anesthetized. Tumor tissues completely resected and harvested for immunohistochemical (IHC) staining or qPCR analysis.

### Immunohistochemical (IHC) staining

Formalin-fixed and paraffin-embedded tissue specimens were prepared for immunohistochemistry. Immunohistochemistry (IHC) staining was performed as described previously [41]. The antibodies used in this study were showed as follows: rabbit Anti-Ki67 antibody (Abcam, ab92742, with 1:500 working dilution), mouse Anti-PHH3 Antibody (Proteintech Group, Inc., CatLog No. 66863-1-Ig, with 1:1000 working dilution).

### Western blotting

Proteins were extracted from cells using RIPA lysis buffer which was mixed with 1% phenylmethanesulfonyl fluoride (PMSF, Jiangsu KeyGEN BioTECH Corp., Ltd, China). BCA method was performed to quantify protein concentration. Protein sample (30 μg per lane) was loaded onto a 10% gel for sodium dodecyl sulfate polyacrylamide gel electrophoresis (SDS-PAGE). Next, the proteins were transferred to polyvinylidene fluoride (PVDF) membranes (Millipore, Burlington, MA, USA), followed by blocking with 5% BSA dissolved in tris buffered saline with tween (TBST, 0.1% [v/v] Tween-20) for 2 h at room temperature. The membranes were then immunoblotted with anti-c-Met antibody (Cell Signaling Technology, Inc., #24294, with 1:1000 dilutions) or anti-β-actin antibody (Cell Signaling Technology, Inc., #4970, with 1:1000 dilutions) overnight at 4 °C. After washing with TBST, the membranes were incubated with anti-rabbit IgG, horseradish peroxidase (HRP)-conjugated antibody (Cell Signaling Technology, Inc., #7074, with 1:8000 dilutions) at room temperature for 2 h. Western blots were detected using the chemiluminescence method.

### Statistical analysis

Data were analyzed using SPSS 22.0 statistical software SPSS (Inc., Chicago, IL, USA). Comparisons between two groups were analyzed by two-sided unpaired *t*-test. Multiple comparisons were analyzed using one-way analysis of variance (ANOVA), post-hoc least significant difference (LSD) test. Differences in Linc00284, miR-27a and c-Met expression between CRC tissue samples and adjacent normal tissues were analyzed using a two-sided paired *t*-test. Chi-square test was used to analyze the clinicopathological characteristics of CRC patients. The overall survival (OS) of patients with colorectal cancer was assessed by Kaplan–Meier curves and the log rank test. Pearson’s correlation analysis was applied to identify the correlation between gene expressions. Receiver operating characteristic (ROC) curves was applied to determine cut-off values to optimize the sensitivity and specificity of Linc00284 and miR-27a [42]. All statistical tests justified as appropriate and the data met the normal distribution. A *P* value less than 0.05 was considered as statistically significant. All data were represented as mean ± standard deviation (SD). An estimate of variation within each group of data was considered and was indicated in each figure as SD. The variance was similar between the groups. Each experiment was repeated independently for three times.

## Supplementary information

Additional Table 1

Figure legends of supplementary figures

Supplementary Figure S1

Supplementary Figure S2

Supplementary Figure S3

Supplementary Figure S4

Supplementary Figure S5

Supplementary Figure S6

Supplementary Figure S7

Supplementary Figure S8

Supplementary Figure S9
