# Supplementary material for: Oncogenic long intervening noncoding RNA Linc00284 promotes c-Met expression by sponging miR-27a in colorectal cancer
Source: Oncogene. 2021 May 28;40(24):4151–66. doi: 10.1038/s41388-021-01839-w (PMC8211564; doi:10.1038/s41388-021-01839-w)
Supplement: Supplementary file 1 — Additional Table 1 [file 41388_2021_1839_MOESM1_ESM.docx]

**Table 1 The list of qPCR primers in this study.**

|  | **Forward Primer** | **Reverse Primer** |
| --- | --- | --- |
| *Linc00284* | 5’-CCAGGGGATAAAACCCGCTT-3’ | 5’-TAAGCACCAAGTCACGCTGT-3’ |
| *U6* | 5’-CTCGCTTCGGCAGCACA-3’ | 5’-AACGCTTCACGAATTTGCGT-3’ |
| *GAPDH* | 5’-TCAACGACCACTTTGTCAAGCTCA-3’ | 5’-GCTGGTGGTCCAGGGGTCTTACT-3’ |
| *miR-27a* | 5’-TTCACAGTGGCTAAGTTCC-3’ | 5’-TCGGCAATTCAGTTGAGC-3’ |
| *miR-27b* | 5’-TTCACAGTGGCTAAGTTCT-3’ | 5’-TCGGCAATTCAGTTGAGC-3’ |
| *Bax* | 5’-CCCGAGAGGTCTTTTTCCGAG-3’ | 5’-CCAGCCCATGATGGTTCTGAT-3’ |
| *Bid* | 5’-ATGGACCGTAGCATCCCTCC-3’ | 5’-GTAGGTGCGTAGGTTCTGGT-3’ |
| *Bcl-2* | 5’-GGTGGGGTCATGTGTGTGG-3’ | 5’-CGGTTCAGGTACTCAGTCATCC-3’ |
| *CyclinD1* | 5’-TGGAGCCCGTGAAAAAGAGC-3’ | 5’-TCTCCTTCATCTTAGAGGCCAC-3’ |
| *CDK4* | 5’-TTCGTGAGGTGGCTTTACTG-3’ | 5’-GATATGTCCTTAGGTCCTGGTCT-3’ |
| *CDK6* | 5’-TCTTCATTCACACCGAGTAGTGC-3’ | 5’-TGAGGTTAGAGCCATCTGGAAA-3’ |
| *Vimentin* | 5’-GCCCTAGACGAACTGGGTC-3’ | 5’-GGCTGCAACTGCCTAATGAG-3’ |
| *E-Cadherin* | 5’-CGAGAGCTACACGTTCACGG-3’ | 5’-GGGTGTCGAGGGAAAAATAGG-3’ |
| *N-Cadherin* | 5’-TCAGGCGTCTGTAGAGGCTT-3’ | 5’-ATGCACATCCTTCGATAAGACTG-3’ |
| *Cytokeratin 19* | 5’-ACCAAGTTTGAGACGGAACAG-3’ | 5’-CCCTCAGCGTACTGATTTCCT-3’ |
| *c-MET* | 5’-AGCAATGGGGAGTGTAAAGAGG-3’ | 5’-CCCAGTCTTGTACTCAGCAAC-3’ |
