# Supplementary material for: Oncogenic long intervening noncoding RNA Linc00284 promotes c-Met expression by sponging miR-27a in colorectal cancer
Source: Oncogene. 2021 May 28;40(24):4151–66. doi: 10.1038/s41388-021-01839-w (PMC8211564; doi:10.1038/s41388-021-01839-w)
Supplement: Supplementary file 2 — Figure legends of supplementary figures [file 41388_2021_1839_MOESM2_ESM.docx]

**Figure legends of supplementary figures**

Figure S1. Overexpression of Linc00284 promotes the proliferation, migration and invasion of CRC cells. (A) The efficiency of Linc00284 overexpression in CRC cells was detected by qPCR analysis after transfection for 48 h. (B) CCT-8 assay was used to detect the cell viability of HCT116 and SW480 cells. (D, E) The proliferative ability of HCT116 and SW480 cells was measured by (D) Ki-67 staining and (E) colony formation assay. (F) The migratory ability of HCT116 and SW480 cells was monitored by wound healing assay. (G) The invasive ability of HCT116 and SW480 cells was monitored by transwell assay. Data are presented as the mean ± S.D from three independent experiments. *P* values were calculated using two-sided unpaired *t*-test. **P*<0.05; ***P*<0.01; ****P*<0.001. OE 00284, overexpression of Linc00284.

Figure S2. Linc00284 and miR-27a are co-expressed in cytoplasm of CRC cells. (A, B) Representative images of the subcellular localization of Linc00284 and miR-27a in (A) HCT116 and (B) SW480 cells. Scale bar, 20 μm.

Figure S3. High transfection efficiency of small interfering RNA (siRNA) to induce Linc00284 silencing in CRC cell lines. (A, B) The silencing efficiency of siRNA that targets Linc00284 in (A) HCT116 and (B) SW680 cells was validated by qPCR analysis after transfection for 5 days. Data are presented as the mean ± S.D from three independent experiments. Multiple comparisons were analyzed using one-way analysis of variance (ANOVA), post-hoc LSD test. **P*<0.05; ***P*<0.01; ****P*<0.001. si, small interfering RNA. si 00284, transient transfection of small interfering RNA to knockdown Linc00284.

Figure S4. The effect of miR-27b on proliferation, migration and invasion of Linc00284 knockdown HCT116 cells. (A) Schematic diagram of the potential binding sites between Linc00284 and miR-27b. (B) Dual-luciferase reporter assay confirmed the interaction between miR-27b and Linc00284. (C) RIP-qPCR analysis revealed that Linc00284 was enriched by probe in CRC cells. (D) RIP-qPCR analysis showed that no enrichment of miR-27b was conformed in CRC cells. (E) The expression levels of miR-27a and miR-27b in CRC cells. (F, G, H) Cell proliferation, migration and invasiveness of Linc00284 knockdown HCT116 cells treated/untreated with miR-27b mimics/inhibitor were measured by (F) colony formation assay and (G, H) transwell assays 48-hour post-transfection. Data are presented as the mean ± S.D from three independent experiments. Comparisons between two groups were analyzed by two-sided unpaired *t*-test. Multiple comparisons were analyzed using one-way analysis of variance (ANOVA), post-hoc LSD test. **P*<0.05; ***P*<0.01; ****P*<0.001.

Figure S5. The effect of Linc00284 overexpression on the expression of c-Met and its downstream genes in CRC cells. (A, B) The expression levels of c-Met and its downstream genes were evaluated by qPCR analysis in Linc00284-overexpressing (A) HCT116 and (B) SW480 cells both with or without miR-27a mimics treatment for 48 h. Data are presented as the mean ± S.D from three independent experiments. *P* value was analyzed using one-way analysis of variance (ANOVA), post-hoc LSD test. **P*<0.05; ***P*<0.01; ****P*<0.001. OE 00284, overexpression of Linc00284.

Figure S6. The effect of Linc00284 knockdown on the expression of c-Met and its downstream genes in CRC cells. (A, B) The expression levels of c-Met and its downstream genes were measured by qPCR analysis in Linc00284-silenced (A) HCT116 and (B) SW480 cells both with or without miR-27a inhibitor treatment for 48 h. Data are presented as the mean ± S.D from three independent experiments. *P* value was analyzed using one-way analysis of variance (ANOVA), post-hoc LSD test. **P*<0.05; ***P*<0.01; ****P*<0.001. sh 00284, lentiviral vector-mediated Linc00284 silencing by short hairpin RNA.

Figure S7. Linc00284/miR-27a axis regulates c-Met expression in CRC cells. The mRNA and protein expression of c-Met in Linc00284-knockdown/overexpressing (A, C, E) HCT116 and (B, D, F) SW480 cells were evaluated by qPCR analysis and western blotting after treatment miR-27a inhibitor, mimics or transfection pcDNA3.1-c-Met plasmid for 48 h. Data are presented as the mean ± S.D from three independent experiments. *P* value was analyzed using one-way analysis of variance (ANOVA), post-hoc LSD test. **P*<0.05, ***P*<0.01, ****P*<0.001. si 00284, transient transfection of small interfering RNA to knockdown Linc00284. OE 00284, overexpression of Linc00284. si c-Met, transient transfection of small interfering RNA to knockdown c-Met. OE c-Met, overexpression of c-Met.

Figure S8. The effect of c-Met inhibitor PHA665752 on c-Met expression in CRC cells. After treatment with c-Met inhibitor PHA665752 (30 ng/ml, 48 h), the mRNA and protein expression of c-Met in Linc00284-knockdown/overexpressing (A, C, E) HCT116 and (B, D, F) SW480 cells were detected by qPCR analysis and western blot assay. Data are presented as the mean ± S.D from three independent experiments. *P* value was analyzed using one-way analysis of variance (ANOVA), post-hoc LSD test. ***P*<0.01; ****P*<0.001; NS, not significant difference. si 00284, transient transfection of small interfering RNA to knockdown Linc00284. OE 00284, overexpression of Linc00284.

Figure S9. Linc00284 affects the responsiveness of CRC cells to hepatocyte growth factor. (A, B) CCK-8 assay was performed to detect cell viability of HCT116 and SW480 cells with linc00284 silencing and HGF treatment (20 ng/ml) for 48 h. (C, D) CCK-8 assay was conducted to test cell viability of HCT116 and SW480 cells with Linc00284 overexpression and HGF treatment (20 ng/ml) for 48 h. Data are presented as the mean ± S.D from three independent experiments. *P* value was analyzed using one-way analysis of variance (ANOVA), post-hoc LSD test. **P*<0.05; ***P*<0.01. HGF, hepatocyte growth factor. si 00284, transient transfection of small interfering RNA to knockdown Linc00284. OE 00284, overexpression of Linc00284.
