# Supplementary figures and images for: Oncogenic long intervening noncoding RNA Linc00284 promotes c-Met expression by sponging miR-27a in colorectal cancer
Source: Oncogene. 2021 May 28;40(24):4151–66. doi: 10.1038/s41388-021-01839-w (PMC8211564; doi:10.1038/s41388-021-01839-w)

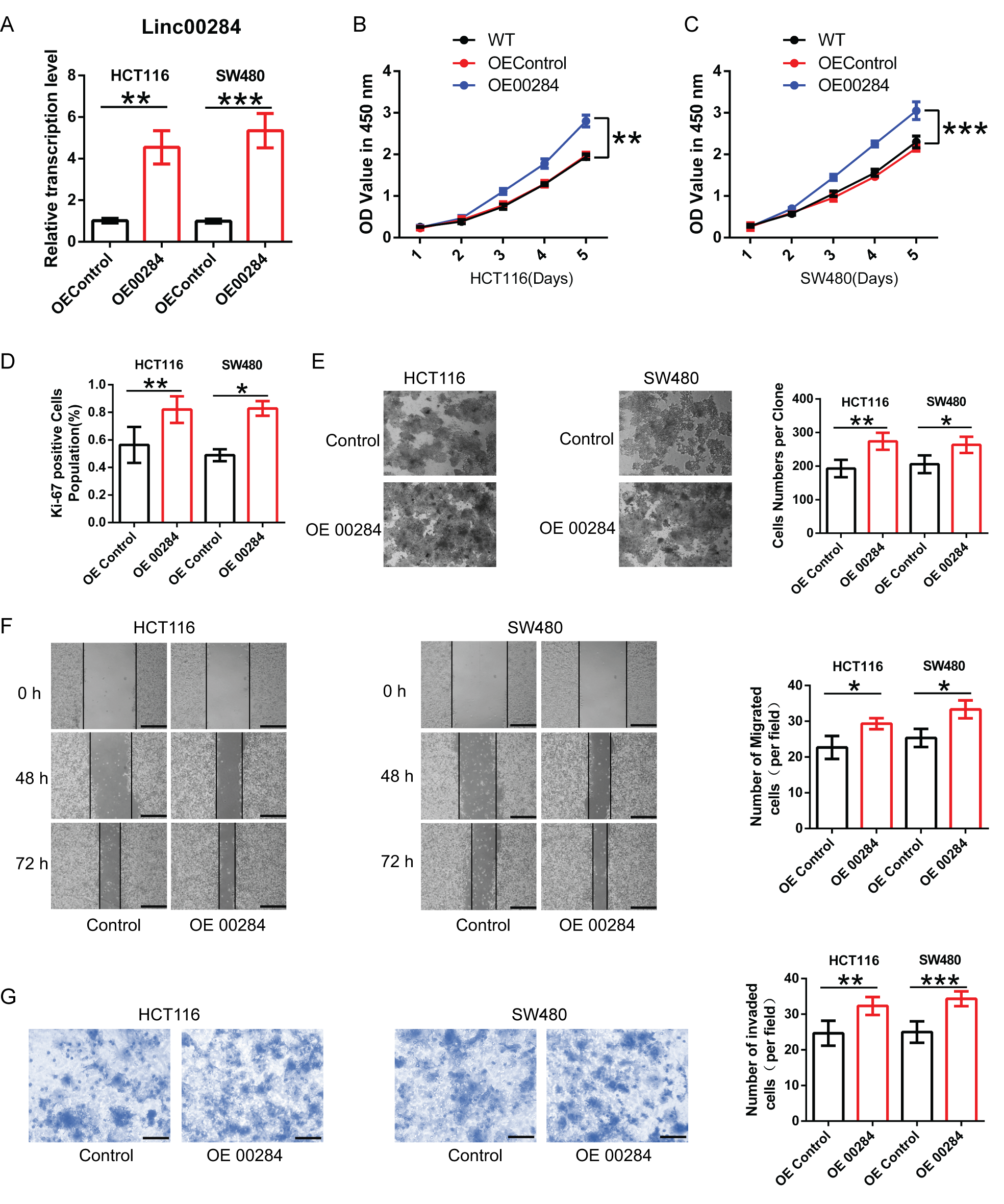

Supplement: Supplementary file 3 — Supplementary Figure S1 [file 41388_2021_1839_MOESM3_ESM.tif]

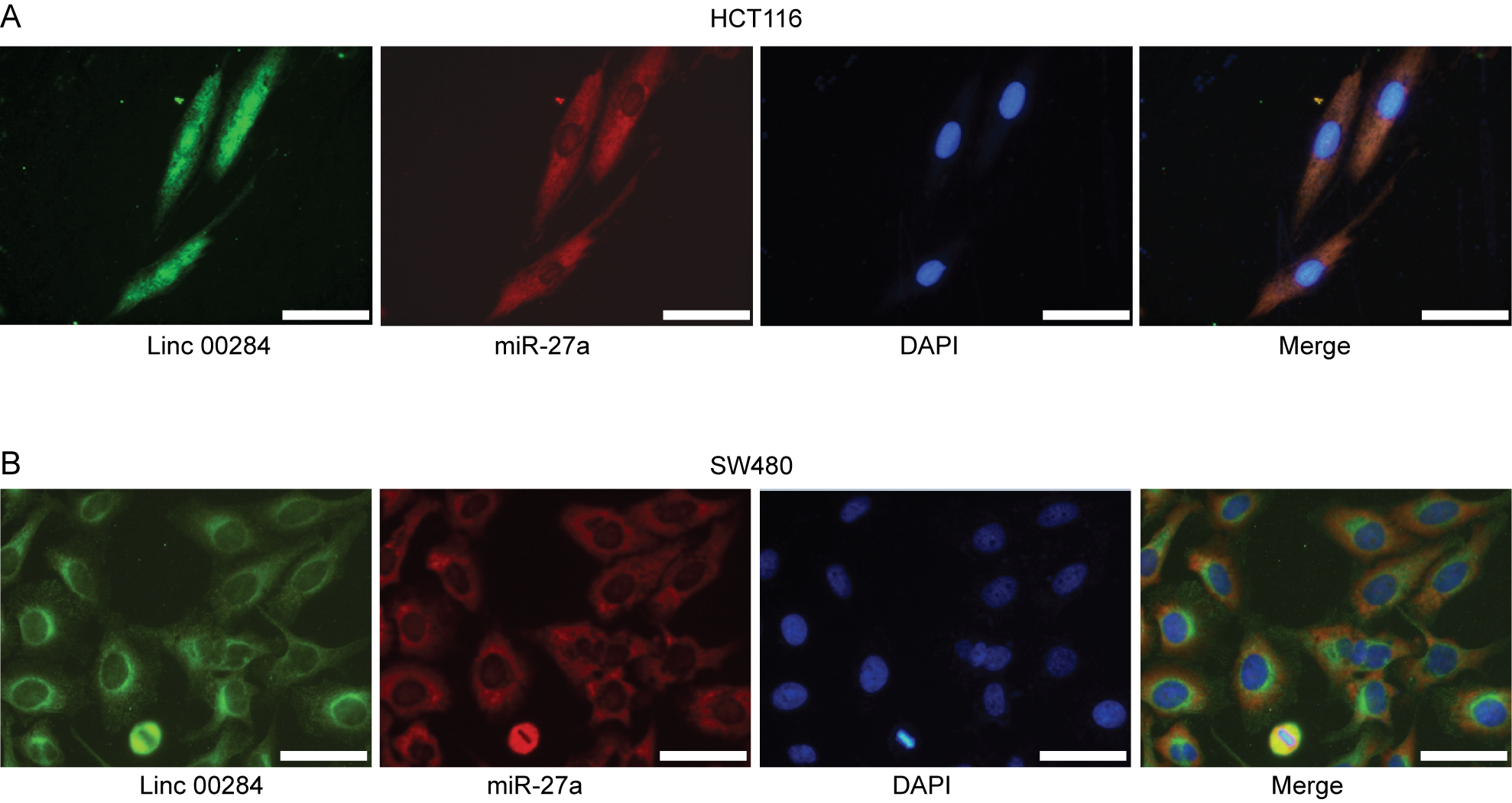

Supplement: Supplementary file 4 — Supplementary Figure S2 [file 41388_2021_1839_MOESM4_ESM.tif]

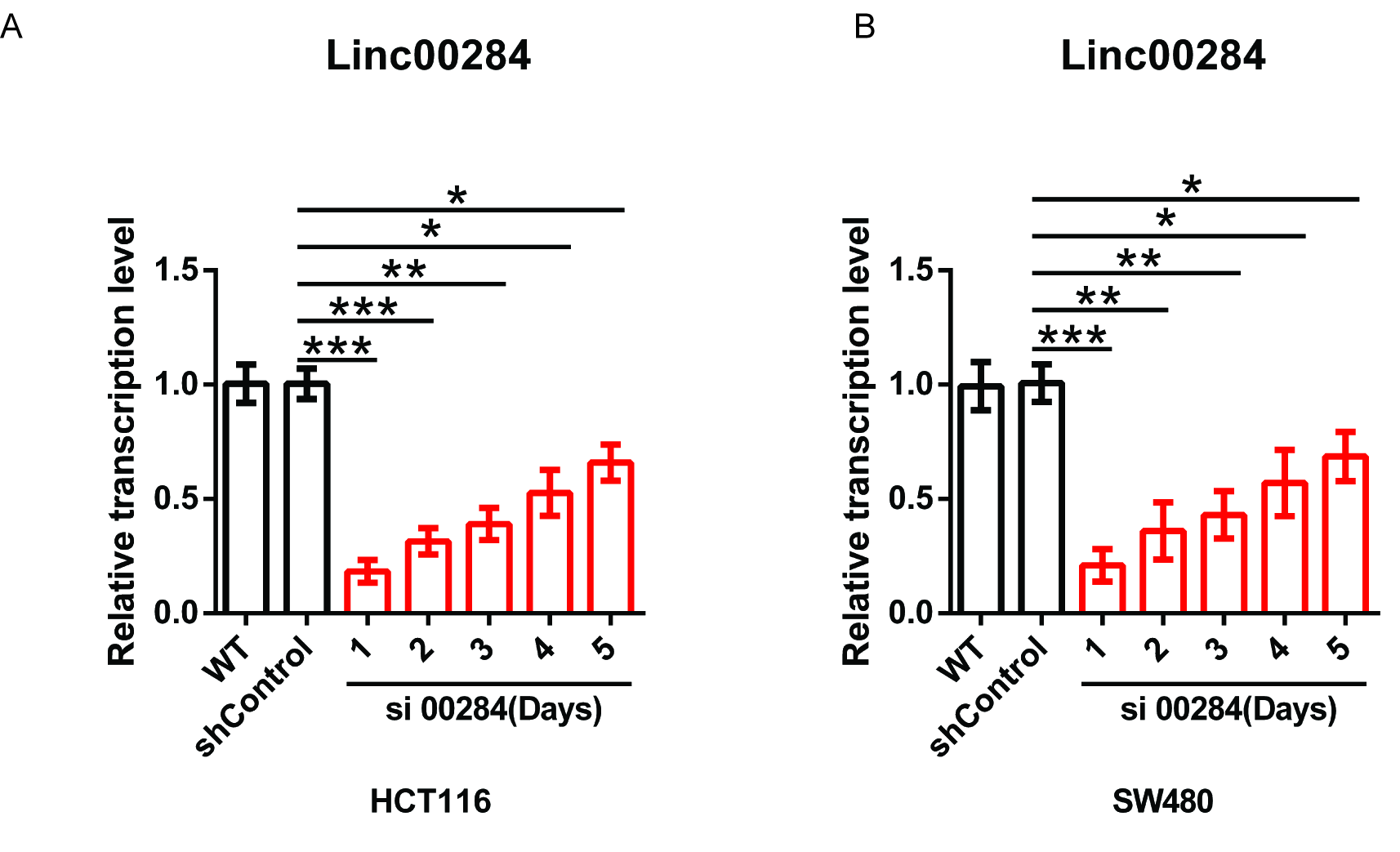

Supplement: Supplementary file 5 — Supplementary Figure S3 [file 41388_2021_1839_MOESM5_ESM.tif]

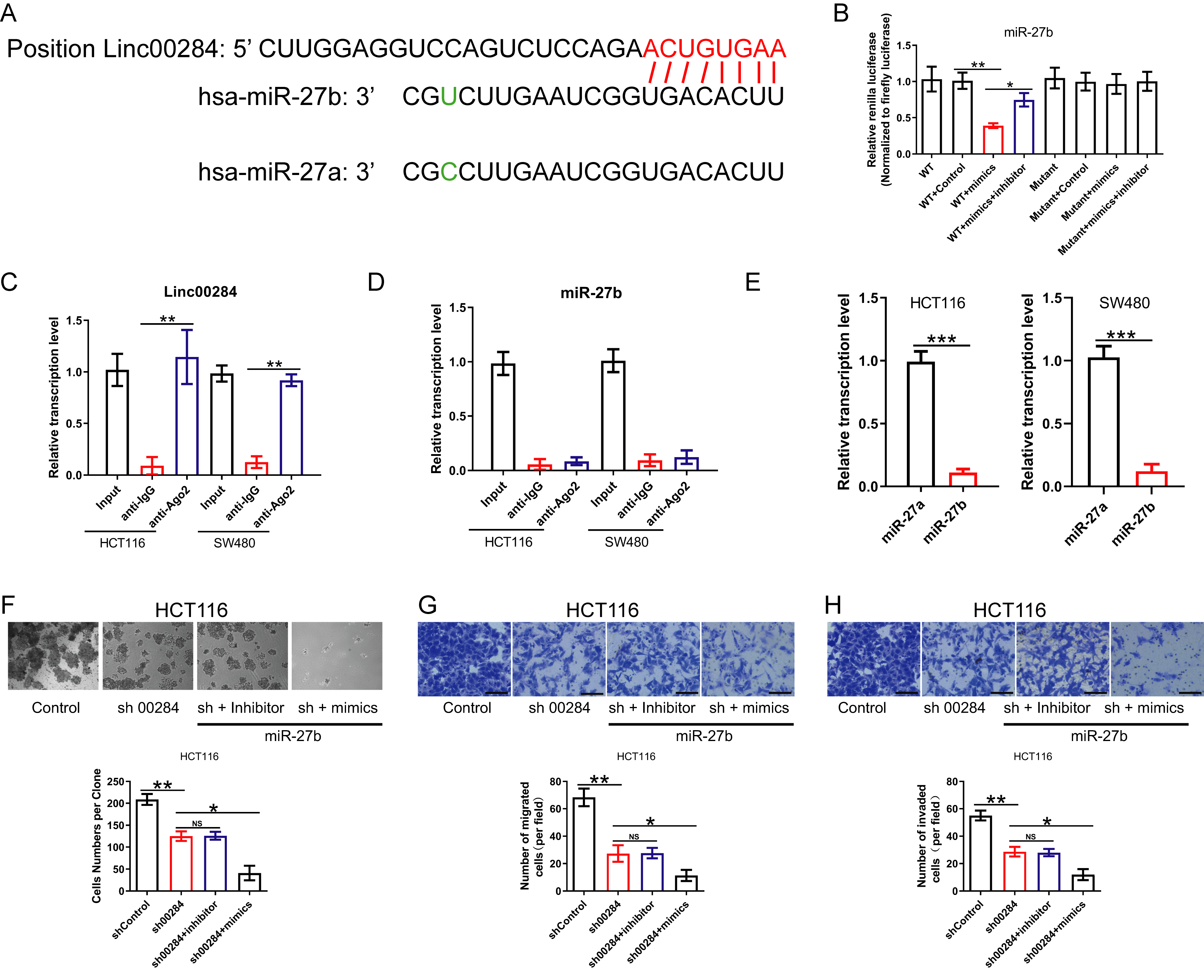

Supplement: Supplementary file 6 — Supplementary Figure S4 [file 41388_2021_1839_MOESM6_ESM.tif]

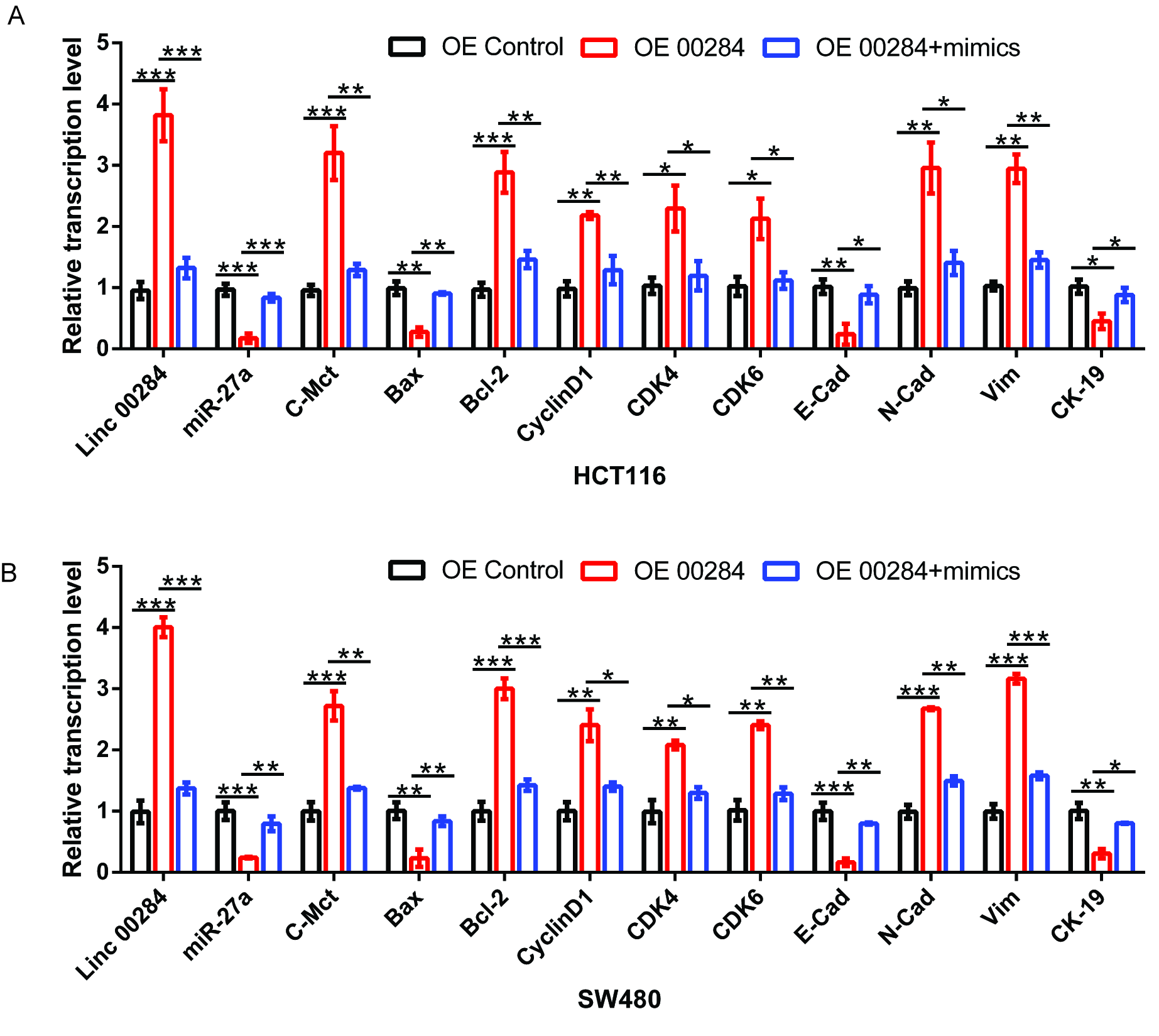

Supplement: Supplementary file 7 — Supplementary Figure S5 [file 41388_2021_1839_MOESM7_ESM.tif]

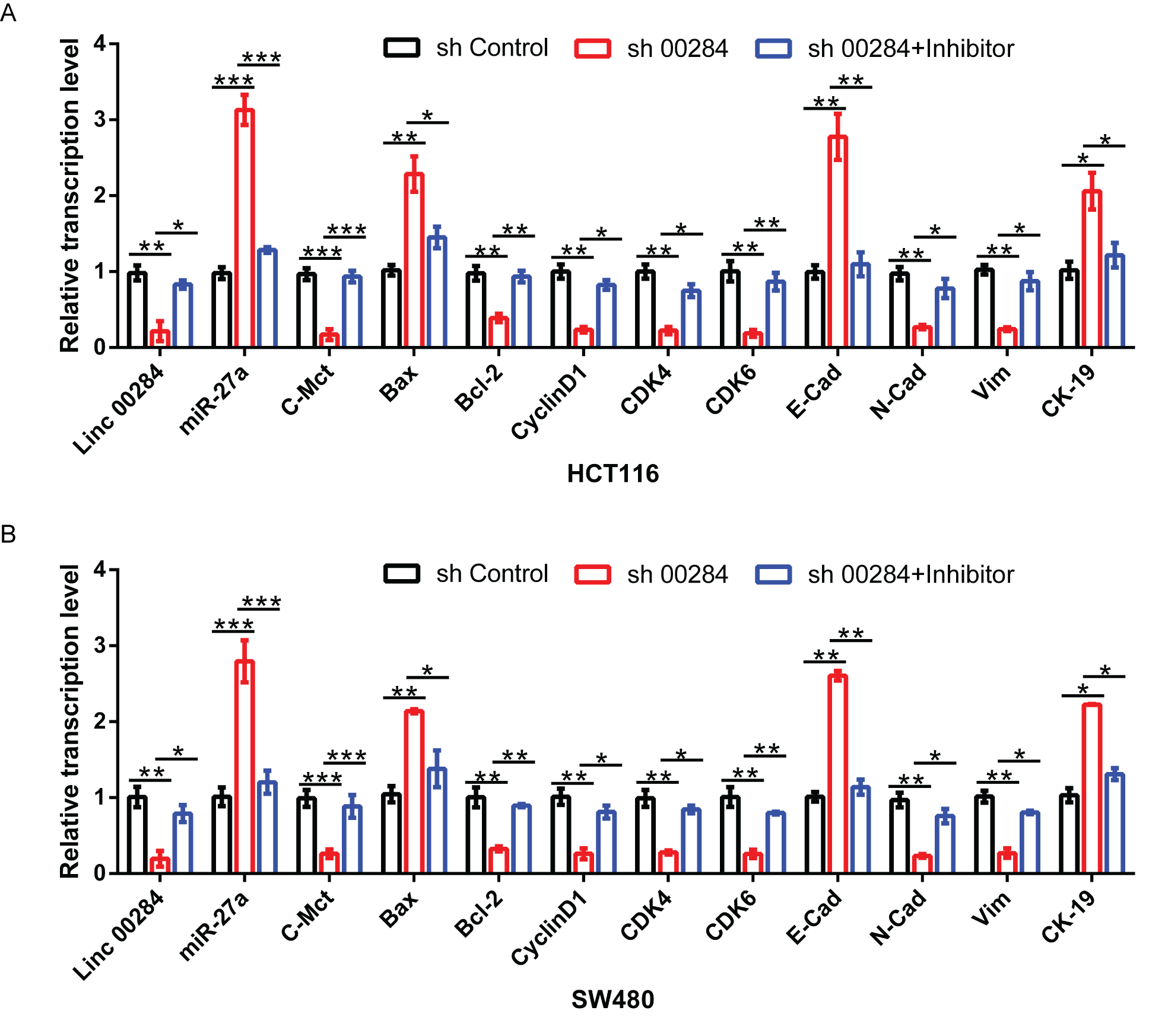

Supplement: Supplementary file 8 — Supplementary Figure S6 [file 41388_2021_1839_MOESM8_ESM.tif]

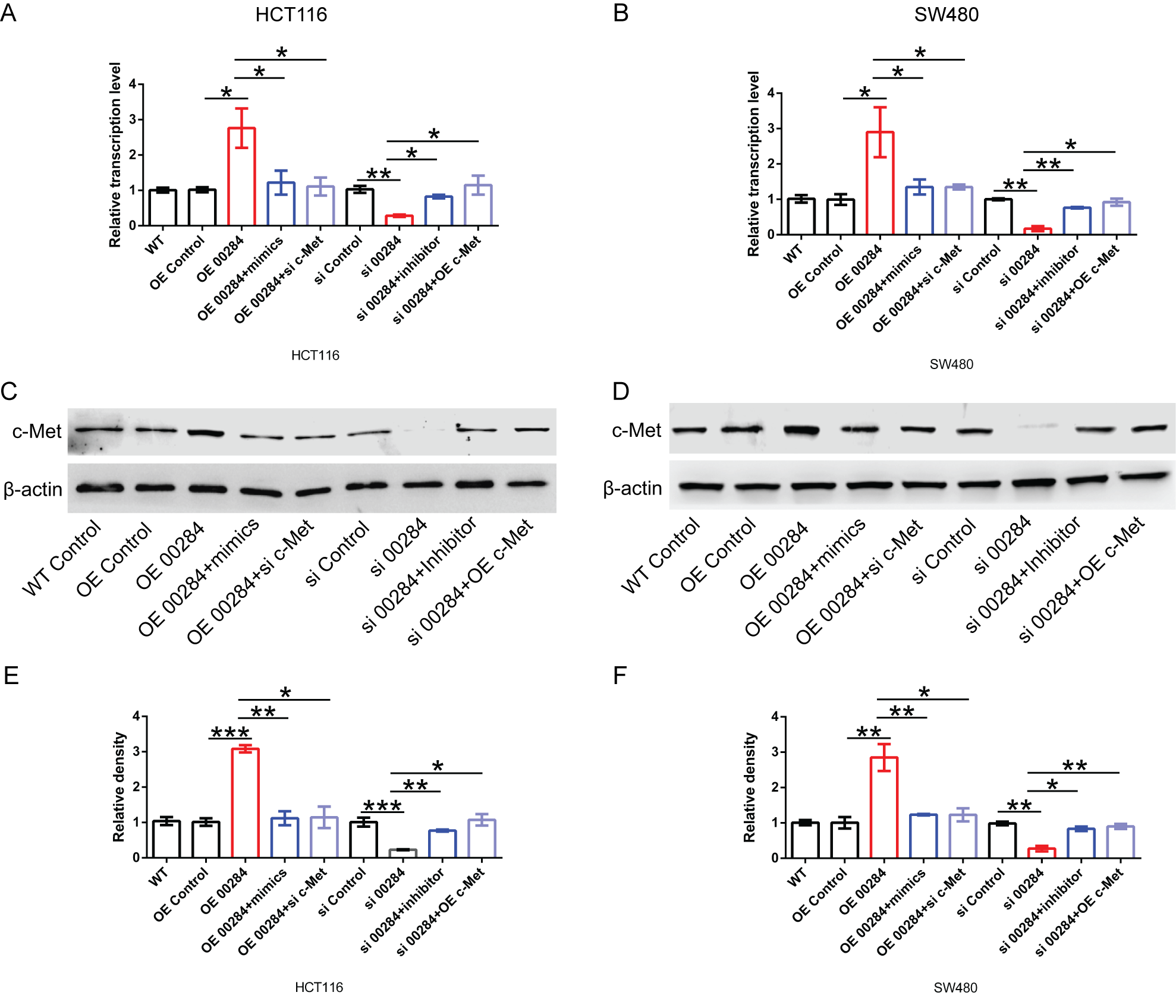

Supplement: Supplementary file 9 — Supplementary Figure S7 [file 41388_2021_1839_MOESM9_ESM.tif]

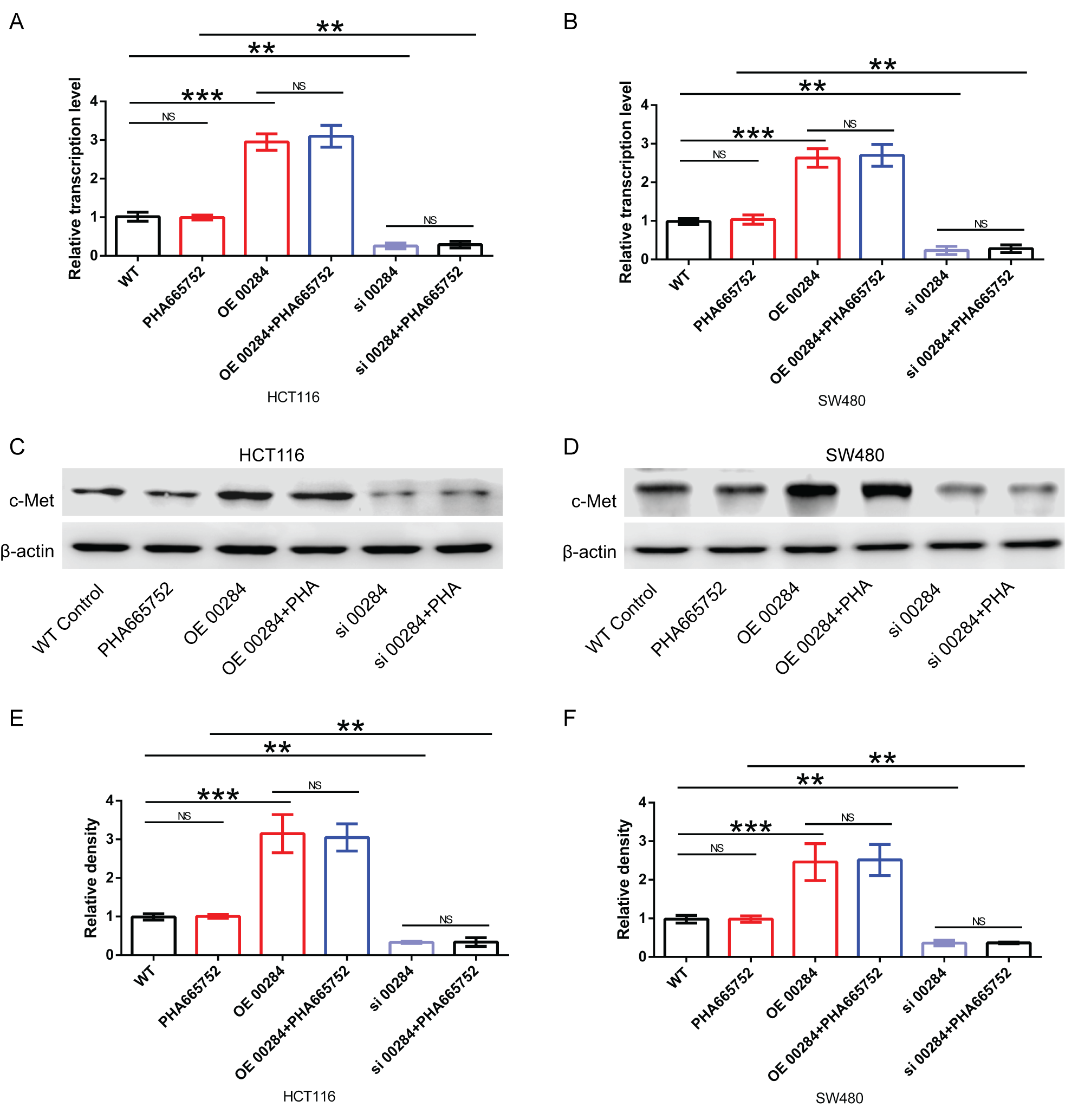

Supplement: Supplementary file 10 — Supplementary Figure S8 [file 41388_2021_1839_MOESM10_ESM.tif]

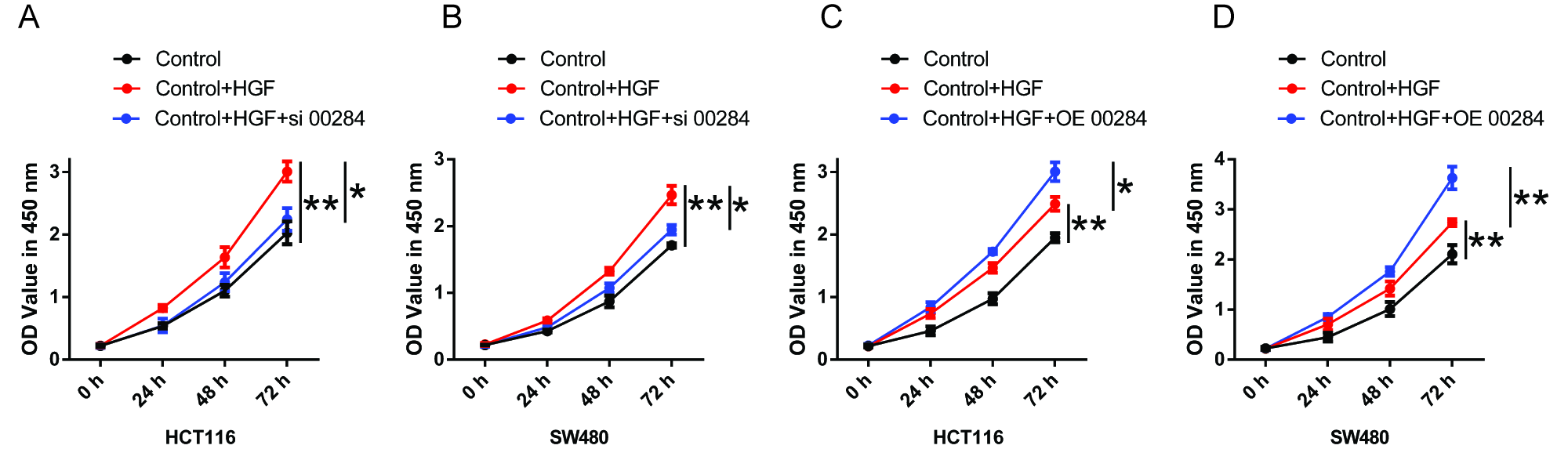

Supplement: Supplementary file 11 — Supplementary Figure S9 [file 41388_2021_1839_MOESM11_ESM.tif]
